# Suppression of piriform cortex activity in rat by corticotropin-releasing factor 1 and serotonin 2A/C receptors

**DOI:** 10.3389/fncel.2015.00200

**Published:** 2015-05-28

**Authors:** Chakravarthi Narla, Henry A. Dunn, Stephen S. G. Ferguson, Michael O. Poulter

**Affiliations:** Molecular Medicine Research Group, Department of Physiology and Pharmacology, Robarts Research Institute, Faculty of Medicine, Schulich School of Medicine, University of Western OntarioLondon, ON, Canada

**Keywords:** piriform cortex, corticotropin-releasing factor, 5-hydroxytryptamine receptors, corticotropin releasing factor 1 receptor, dimethoxy-4-iodoamphetamine, adapting high frequency, adapting low frequency, non-adapting very high frequency

## Abstract

The piriform cortex (PC) is richly innervated by corticotropin-releasing factor (CRF) and serotonin (5-HT) containing axons arising from central amygdala and Raphe nucleus. CRFR_1_ and 5-HT_2A/2C_Rs have been shown to interact in manner where CRFR activation subsequently potentiates the activity of 5-HT_2A/2C_Rs. The purpose of this study was to determine how the activation of CRFR_1_ and/or 5-HT_2_Rs modulates PC activity at both the circuit and cellular level. Voltage sensitive dye imaging showed that CRF acting through CRFR_1_ dampened activation of the Layer II of PC and interneurons of endopiriform nucleus. Application of the selective 5-HT_2A/C_R agonist 2,5-dimethoxy-4-iodoamphetamine (DOI) following CRFR_1_ activation potentiated this effect. Blocking the interaction between CRFR_1_ and 5-HT_2_R with a Tat-CRFR_1_-CT peptide abolished this potentiation. Application of forskolin did not mimic CRFR_1_ activity but instead blocked it, while a protein kinase A antagonist had no effect. However, activation and antagonism of protein kinase C (PKC) either mimicked or blocked CRF modulation, respectively. DOI had no effect when applied alone indicating that the prior activation of CRFR_1_ receptors was critical for DOI to show significant effects similar to CRF. Patch clamp recordings showed that both CRF and DOI reduced the synaptic responsiveness of Layer II pyramidal neurons. CRF had highly variable effects on interneurons within Layer III, both increasing and decreasing their excitability, but DOI had no effect on the excitability of this group of neurons. These data show that CRF and 5-HT, acting through both CRFR_1_ and 5-HT_2A/C_Rs, reduce the activation of the PC. This modulation may be an important blunting mechanism of stressor behaviors mediated through the olfactory cortex.

## Introduction

In the central nervous system, CRF binds with high affinity to CRF_1_ receptors (CRFR_1_) but with only moderate affinity to CRFR_2_ (Zorrilla et al., 2014). CRF within CNS originates from cell bodies in central amygdala as well as interneurons in the cortex (Anisman et al., 2007). CRFR_1_ receptor activation has been shown to have a number of differing effects on cellular behavior that varies with brain region. In prefrontal cortex it prolongs the serotonin (5-HT) induced increase in GABAergic synaptic activity (Tan et al., 2004). While in CA1 pyramidal neurons it modulates potassium currents (A and delayed rectifier types; Kratzer et al., 2013). It may also dampen excitability by inhibiting NMDA-induced currents in cultured hippocampal neurons (Sheng et al., 2008). In the amygdala CRF activates a Kv3 type potassium channels that contribute to action potential repolarization therefore slowing firing frequency (Fu and Neugebauer, 2008). Thus the effects of CRF seem to be highly diverse. This varied functionality may arise from heterogeneity of G proteins that couple to CRF receptors. For example, in above mentioned studies these actions were mediated by either protein kinase A (PKA; Fu and Neugebauer, 2008) or protein kinase C (PKC; Tan et al., 2004) signaling pathways.

A similar situation is apparent for the neurotransmitter 5-HT which arise from cells located in the Raphae nucleus (Sheldon and Aghajanian, 1990). Its activity throughout the brain is highly diverse. For example, Aghajanian and co-workers found that 5-HT is excitatory on interneurons and inhibitory on pyramidal cells of PC (Sheldon and Aghajanian, 1990, 1991; Marek and Aghajanian, 1996). However, unlike CRF, this diversity in activity of 5-HT seems to be due to the wide range of 5-HT receptor subtypes that are heterogeneously expressed throughout the brain.

The heterogeneous functions of both these neurotransmitters are thought to be important in mediating stressor or anxiety responses (Eison, 1990; Hayley et al., 2005). For example, it has been well-documented that DOI, a 5-HT_2A/C_R agonist produces very strong anxiety responses, and increases in extrahypothalamic CRF has been associated with anxiety disorders (Anisman et al., 2008; Magalhaes et al., 2010). CRFR_1_ activation can enhance 5-HT_2A/C_R mediated signaling via a mechanism involving the recruitment of endosomal 5-HT_2A/C_R to the plasma membrane (Magalhaes et al., 2010). Furthermore the same study showed that prior dosing of mice with a CRFR_1_ agonist enhanced DOI-induced anxiety. However, an understanding of how these neurotransmitters affect the neural circuit behavior is incomplete and more research is needed to understand the impact of their activation on local circuit behavior.

One region of the brain that highly expresses both CRFR_1_ and 5-HT_2_Rs is the PC (Pazos et al., 1985; Van et al., 2000). The PC is of interest in epilepsy because its circuitry easily supports seizures (Ekstrand et al., 2001). We have recently shown that PC excitability is under the control of feed forward disinhibitory circuit that potentiates excitatory input from the lateral olfactory tract (LOT; Birjandian et al., 2013). How CRF and/or 5-HT may modulate this circuit is not known. Therefore, the purpose of this study was to understand how CRFR_1_ activation and their possible interaction of 5-HT_2A/C_R modulate the activity of the PC.

## Materials and Methods

All the procedures performed for this project were in accordance with the guidelines of Canadian council of animal care and approved by The University of Western Ontario council on animal care.

### Slice Preparation and Dye Loading

Adult male Sprague-Dawley rats weighing 150–180 g were used in all experiments. They were housed individually with free access to food and water under a continuous 12 h light/dark cycle. Animals were anesthetized with ketamine–medetomidine hydrochloride combination and then perfused through heart with an ice-cold artificial cerebrospinal fluid (ACSF) in which sodium ions was replaced by choline ions. The composition of this ACSF used is choline chloride, 110 mM; KCl, 2.5 mM; NaH_2_PO_4_, 1.2 mM; NaHCO_3_, 25 mM; CaCl_2_, 0.5 mM; MgCl_2_, 7 mM; sodium pyruvate, 2.4 mM; ascorbate, 1.3 mM; dextrose, 20 mM (McIntyre et al., 2002). The brains were perfused to flush any blood out of the vessels to prevent the iron in the blood from oxidizing and causing damage to neuronal cells. The brain was rapidly removed following perfusion and the region containing anterior PC was carefully cut into a block to facilitate slicing by a Vibratome (slices were 400 μM thick). The slices were incubated at room 37°C for 30 min and subsequently moved to room temperature (22°C) bath for 45 min. The perfusion, slicing and incubation procedures were carried out in choline-ACSF with continuous supply of carbogen (95% O_2_ and 5% CO_2_ mixture). These slices were used for both voltage sensitive dye imaging (VSDI) and patch clamp recording. For the VSDI slices were incubated in the voltage sensitive dye Di-4-ANEPPS (D-199, Invitrogen Molecular Probes, Inc., Eugene, OR, USA) for 35 min. The stock solution of the dye was dissolved in ethanol (22 mg/ml). On the day of experiment the dye incubation was prepared by mixing 60 μl of dye stock with 500 μl of fetal bovine serum (FBS), 500 μl of ACSF and 310 μl of 10% cremophore-EL solution. The concentration of dye in the final solution was 0.1 mg/ml. After incubation slices were washed for 8–10 min with ACSF and transferred to recording chamber. The temperature of the bath was maintained at 32°C during recordings and continuously supplied with carbogen bubbled ACSF having a composition of NaCl, 110 mM; KCl, 2.5 mM; NaH_2_PO_4_, 1.2 mM; NaHCO_3_, 25 mM; CaCl_2_, 2.0 mM; MgCl_2_, 2.0 mM; dextrose, 20 mM. The pH and osmolarity of the solutions were adjusted to 7.3–7.4 and 297–305 mOsm respectively.

A platinum/iridium electrode (Microprobes, Inc., Gaithersburg, MD, USA) with a tip diameter of 200–300 μM was used to stimulate LOT of PC. The stimulation of each slice was in the range of 160–200 μA, each square pulse was 2.0 ms in length. The electrode was connected to a stimulator (S88X dual output square pulse stimulator, Grass Technologies, Astro-Med, Inc., Brossard, QC, Canada), which controlled the pulse frequency and train duration (Birjandian et al., 2013).

### Voltage Sensitive Dye Imaging

Each optical recording was about 10 s in length and consisted of two phases. The first contained recording of background activity for 2 s followed by the application of stimulus for 1 s with a train of frequencies ranging from 5 to 80 Hz. The acquisition rate was set at 5 ms/frame. The camera saturation was set around 50% for each recording. Optical signals were recorded by a CMOS camera (Micam Ultima Brain Vision, Inc., Tokyo, Japan) mounted on top of an upright microscope (Fixed stage upright microscope, BX51WI, Olympus). The light from a 100 W halogen lamp source (HLX 64625, Microlites Scientific, Corp.) passed through an excitation filter (λ = 530 ± 10 nm). A long pass emission filter (λ > 590 nm) collected the fluorescent signals. A long working distance objective was used in the experiments (XLFluor 4X N.A.0.28, Olympus). The movies were analyzed using Brain Vision Analyzer (Tokyo, Japan) software. A detailed explanation of the technique is described elsewhere (Birjandian et al., 2013).

### Patch Clamp Electrophysiology

Patch clamp recordings were done as previously described in McIntyre et al. (2002) and Gavrilovici et al. (2012). Patch electrodes were pulled from borosilicate glass capillaries and filled with K^+^-gluconate solution having a composition (in mM) of: potassium gluconate 147, KCl 1, CaCl_2_ 2, HEPES 10, EGTA 10, Glucose 10, MgATP 2, GTP 0.3 (300 mOsm, pH 7.3–7.4). Whole-cell patch clamp recordings were made with an Axon instruments Patch 700B amplifier (Molecular Devices, Sunnyvale, CA, USA) from neurons in Layers II and III of anterior PC. Series resistance compensation was performed in all recordings. The initial access resistance was <20 MΩ and compensated by 50–70%. Bridge balance was set to auto to correct the voltage drop across the membrane. All experiments were performed at 32°C. Stimulation of the LOT for these recordings was done in an identical manner as that used for VSDI. Excitability of the cells was assayed by current-clamp protocols (Axon instruments; Clampex 10.3; 500 ms pulses in 50 pA increments). For recordings done in Layer III we have previously identified by the cluster analysis five differing functional phenotypes (Gavrilovici et al., 2012). These clusters were named according to the terminology described by Kroner et al. (2007) and Ascoli et al. (2008). To decide whether interneuron significantly slowed action potential generation (adapted) an interspike interval ratio (II_R_) was calculated. The II_R_ is the ratio between the last spike interval at the end of the 500 ms current pulse divided by the first spike interval. An II_R_ < 1.25 were termed as non-adapting cells while neurons with II_R_ > 1.25 were classified as adapting cells. As well cells were classified based on their firing frequency (FF) at 2X current threshold (I_2t_). Cells that had an average FF > 50 Hz were classified as high frequency (HF) while those FF < 50 Hz were termed as low frequency (LF). Cells with a FF of >100 Hz were classified as very high frequency (vHF).

### Tat Peptide Co-immunoprecipitation

Human embryonic kidney (HEK293) cells were maintained in Eagle’s minimal essential medium with 10% FBS. HEK293 cells were seeded onto 10 cm dishes 24 h before transfection to attain 70–80% confluency. Transient transfection was performed using a modified calcium phosphate protocol described by Ferguson and Caron (2004). Cells were transfected with 1 μg of YFP-SAP97 and 1 μg of either FLAG-5-HT_2A_R or HA-CRFR_1_. These constructs have been described previously (Dunn et al., 2013, 2014). 18 h post-transfection, cells were washed with phosphate-buffered saline (PBS) and re-suspended with trypsin containing 0.25% EDTA. 24 h after the removal of transfection reagents, cells were treated with Tat-Scrambled or Tat-CRFR_1_-CT for 2 h at the described concentrations (Tao et al., 2008). Cells were subsequently lysed in lysis buffer (50 mM Tris, pH 8.0, 150 mM NaCl, and 1% Triton X-100) containing protease inhibitors (1 mM AEBSF, 10 μg/ml leupeptin, and 5 μg/ml aprotinin) for 20 min on a rocking platform at 4°C. Samples were collected into 1.5-ml Eppendorf tubes and centrifuged at 15,000 × *g* for 15 min at 4°C to pellet insoluble material. A Bronsted–Lowry protein assay was performed, and 400 μg of protein from each condition was incubated for 1–2 h at 4°C with either FLAG-immunoprecipitation beads from Sigma-Aldrich or protein G-sepharose and mouse anti-HA antibody (1:50). After incubation, beads were washed three times with cold lysis buffer and incubated overnight at room temperature in 3x SDS Loading Buffer containing 2-mercaptoethanol. Samples were separated by SDS-PAGE, transferred to a nitrocellulose membrane, and immunoblotted to identify co-immunoprecipitated YFP-SAP97 (rabbit anti-GFP, 1:1000) with either FLAG-5-HT_2A_R or HA-CRFR_1_ in the presence or absence of Tat-CRFR_1_-CT.

### Reagents

2,5-Dimethoxy-4-iodoamphetamine, H-89, Forskolin and BIS were obtained from Sigma-Aldrich Co., St. Louis, MO, USA. CRF, antisauvagine-30 and antalarmin were obtained from Tocris Biosciences, Ellisville, MO, USA. Tat-CRFR_1_-CT peptide was obtained from CanPeptide, Inc., Pointe-claire, QC, Canada. The Tat-CRFR_1_-CT sequence used was YGRKKRRQRR-PTRVSFHSIKQSTAV. This peptide has been shown previously to prevent the functional synergism between CRFR_1_ and 5-HT_2_Rs (Magalhaes et al., 2010). Di-4-ANEPPS stock solution was prepared in alcohol and cremophore-EL solution which can be stored at 4°C for 2 months. FBS and ACSF were added on the day of experiment. CRF stock was dissolved in HBSS and milli-Q water mixture. DOI was prepared in alcohol and Milli-Q water. Forskolin, H-89, BIS, antalarmin, antisauvagine-30, and PMA stock solutions were prepared in dimethyl sulfoxide (DMSO). All the stock solutions were made 1000 times more concentrated than working concentrations.

### Statistical Analysis

Comparison of all measured values was done using two-way analysis of variance (ANOVA) or repeated measures ANOVA as appropriate. *Post hoc* comparisons were carried out using a Fisher Exact Test. A Mann–Whitney test (non-parametric) was used to evaluate the action potential number data taken form pyramidal cell recordings. The significance for all tests was set at *p*< 0.05. All statistical evaluations were done using Statview Software.

## Results

**FIGURE 1:**
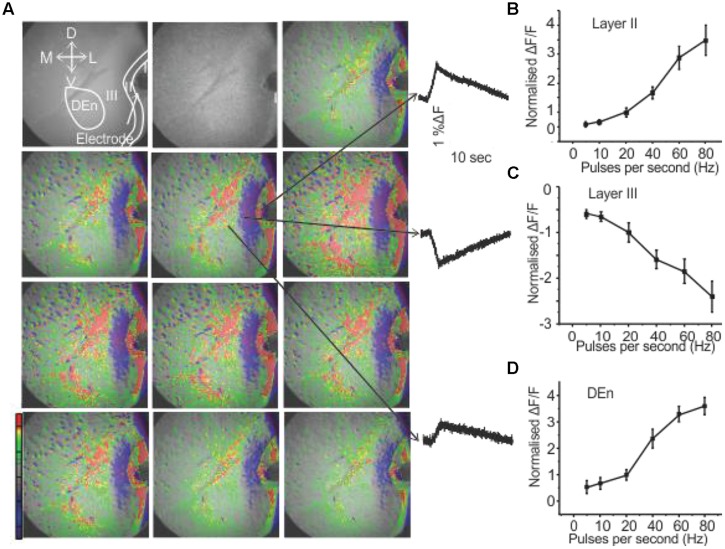
**Activation of PC involves three kinds of responses originating in different anatomical regions. (A)** Show a 10 s long recording where a 80 Hz stimulation was used (red, green, yellow represents depolarization: blue, magenta, violet represent hyperpolarization). Waves on the right side indicated by arrows represent depolarization responses from Layer II and dorsal endopiriform nucleus (DEn) and a hyperpolarizing response from Layer III (arrows in the first panel represent the orientation of the slice. D: dorsal; V: ventral; M: medial; L: lateral). **(B–D)** Graphs of normalized change in ΔF/F vs. stimulus intensity from nine recordings in the Layers II, III and DEn respectively. Activation of each response increased over the range of stimulation of 20–80 Hz (γ-range).

We have recently demonstrated that the activation of the PC through the stimulation of the LOT induces a novel feed forward disinhibitory loop that potentiates activation of the Layer II pyramidal cell layer (see Birjandian et al., 2013) for a complete description). Responses were followed by VSDI of the PC brain slices (sampling rate is 200 frames/second) that include all three layers along with the underlying dorsal endopiriform nucleus (DEn). When the (LOT) was stimulated with a bipolar electrode with frequencies ranging from 20 to 80 Hz for 1 s Layer II was first activated. This initial response was followed about 40–50 ms later by the activation of the dorsal endopiriform nucleus (DEn) and then (after about 200 ms) the deactivation of Layer III. An example of a response is shown in **Figure 1**. The first frame, before the stimulation, shows of the PC Layers I–III and endopiriform nucleus (DEn). Each subsequent frame in the picture has a time difference of 2 s from the preceding frame. The excitatory responses originating from Layer II and DEn are shown in **Figures 1B,D**. The inhibitory response originating from Layer III is shown in **Figure 1C**. The change in fluorescence in these recordings is color coded by the software so that excitation is shown by green, yellow, and red while decreased activity is shown by blue and magenta colors. Our interpretation of this sequence of events is that the initial activation of the Layer II activates inhibitory neurons in the DEn that then in turn inhibit Layer III neurons. This disinhibition of Layer III potentiates the activation of Layer II neurons (again see Birjandian et al., 2013) for complete characterization of this response). In **Figures 1B–D** we show the relationship between frequency of stimulation and change in the magnitude of the response in Layers II, III, and DEn respectively showing that the (de)activation occurs over the gamma frequency band of stimulation trains.

### CRF Reduces the Activity of the PC

**FIGURE 2:**
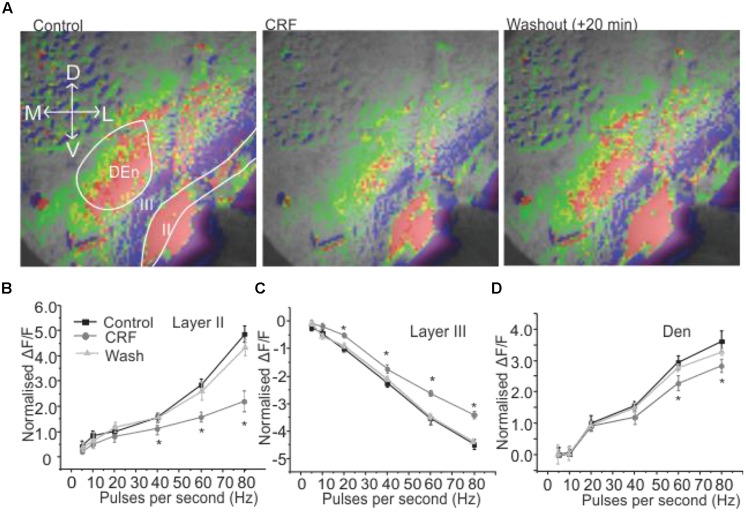
**Activation of CRFR_1_ in PC reduced the activation of pyramidal cell layer (Layer II) and dorsal endopiriform nucleus (DEn) but attenuated the deactivation of interneuronal layer (Layer III)**. Representative images taken 1 s after the end of stimulation are shown in **(A)** before (left) and after the application of 100 nM CRF (middle 15 min) and after 20 min wash (right). In **(B–D)** we show quantification of CRFR_1_ effects over the range of stimulation frequencies used to activate the circuit. CRF was most effective on the 60 and 80 Hz stimulations. ^*^p < 0.05.

We wanted to investigate how CRF might modulate this response. This was done by comparing control input/output relationship (as shown in **Figures 1B–D**) to those obtained after ACSF containing 100 nM CRF was perfused over the slice. In **Figure 2A**, the left panel shows the control response (2 s after the stimulation) to an 80 Hz train. The middle panel shows the equivalent response after 15–20 min of 100 nM CRF application and the right most panel shows the response after CRF washout. CRF reduced the activity of pyramidal cell layer as evidenced by decreased signal (less red) in Layer II [*F*(1,9) = 6.58, *p* < 0.001, *n* = 9]. Similarly, the activity of neurons in DEn was also reduced which can be seen by decreased green/yellow/red scaling of the fluorescence signal. The decreased blue/violet color in Layer III represents decreased disinhibitory drive arising from the DEn neurons. In **Figures 2B–D**, we show the effect of CRF over the entire input/output relationships in the three areas tested. CRF reduced the activity of Layer II pyramidal cells and the cells in DEn as the curves shifted downward (**Figures 2B,D**). Application of CRF also reduced the inhibition of Layer III, thus shifting the curve upward. This CRF effect was reversible within 10 min (**Figure 2C**). We also found that the effects of CRF were most robust within γ-frequency range (40–80 Hz). There was no significant difference in fluorescence signals within θ to high β frequency range (5–20 Hz).

### CRFR_1_ Mediate the Actions of CRF in PC

**FIGURE 3:**
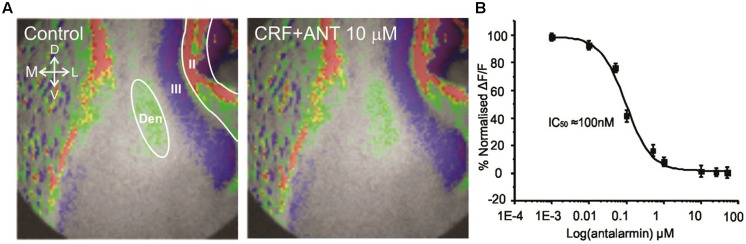
**The effects of CRF are blocked by the CRFR_1_ antagonist antalarmin**. In **(A)** we show a representative image of the control recording (on the left). The image on the right shows that in presence of 10 μM antalarmin CRF was without effect on the activation of the PC circuitry. In **(B)** we show dose inhibition curve for CRFR_1_ receptor blockade in Layer II of PC over the range of antalarmin concentrations used. The IC_50_ for antalarmin was found to be 100 nM.

Corticotropin-releasing factor exerts its actions through two major kinds of receptor subtypes named CRFR_1_ and CRFR_2_ (Zorrilla et al., 2014). These two receptors are widely distributed in the rat central nervous system (De Souza et al., 1985; Primus et al., 1997; Van et al., 2000). Since these receptors bind to different G proteins, activate various signaling cascades and produce different effects it is essential to understand which CRFR subtype mediate the actions of CRF in this context. We employed antalarmin, a CRFR_1_ antagonist to see if it blocks the actions of CRF. We found that antalarmin at a concentration of 10 μM abolished the activity of CRF [100 nM; *F*(1,6) = 6.542, *p* < 0.001, *n* = 10]. In **Figure 3A** we show that there is no difference in control (left panel) and CRF and antalarmin treated slice (right panel). In **Figure 3B**, we show the dose/inhibition curve of antalarmin in the presence of 100 nM CRF. We found that the IC_50_ of antalarmin was 100 nM. We also tested antisuvagine-30, a CRFR_2_ antagonist to determine whether CRFR_2_ has any role in the actions of CRF. Antisauvagine-30 was not able to block the actions of CRF (data not shown). These data indicate that CRF reduces the excitability of the PC through the activation of CRFR_1_.

### Induction of DOI Activity is Dependent on Previous CRF Activation of CRFR_1_

**FIGURE 4:**
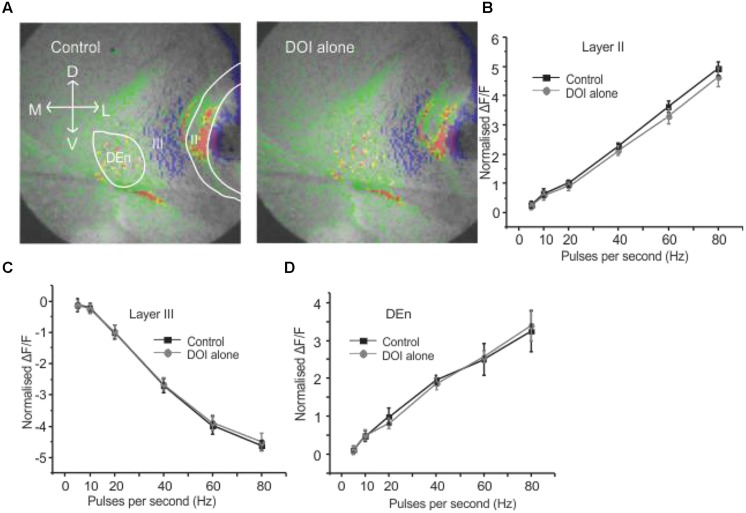
**Activation of serotonin (5-HT) receptors alone had no effect on PC circuitry**. Representative images in **(A)** were taken before and after application of DOI, a 5-HT_2A/2C_R agonist. In **(B–D)** we show that DOI was without effect in each region followed over the entire range of stimulation frequencies used to activate the circuit.

**FIGURE 5:**
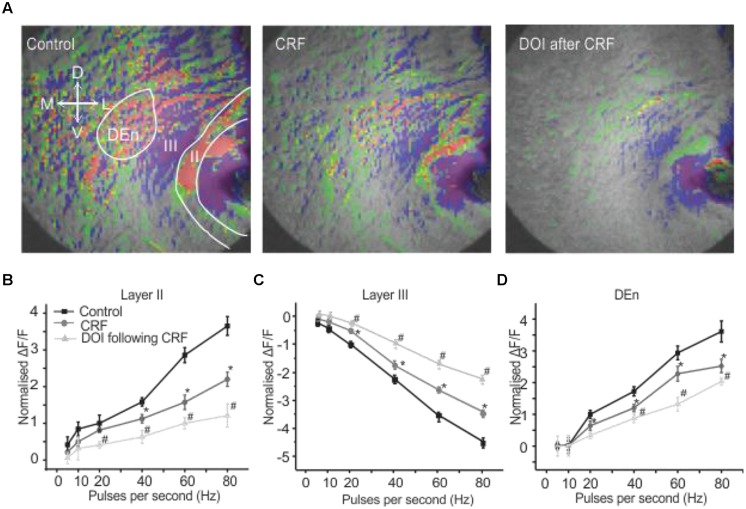
**Activation of CRFR_1_ before the application of DOI potentiated the CRF effects**. Representative images in **(A)** show control (left), after the application of CRF (middle) and the subsequent application of DOI following CRF (right). The image on right was taken after 15 min of perfusion with CRF. In **(B–D)** we show quantitation of CRFR_1_ and 5-HT_2A/C_R activation over the range of stimulation frequencies used to activate the circuit. ^*^#*p* < 0.01. ^*^CRF effect is significantly different compared to control. # DOI following CRF effect is significantly different from CRF. DOI was immediately applied after the CRF application was stopped. However, there was at least 15–17 min time lapse between when the DOI application was started and the slice was stimulated for the recording. The perfusion bath emptying rate was maintained at 1 ml per min. When DOI was added in the absence of CRF.

It has been previously reported that CRFR_1_ potentiates 5-HT_2_R mediated signaling *in vitro* (Magalhaes et al., 2010). Therefore, we wished to see if these receptors could alter PC activation and perhaps act in similar synergistic manner. We used the relatively selective 5-HT_2A/C_ receptor agonist DOI. Surprisingly, DOI had no effect on the PC circuit activation (*n* = 5). **Figure 4A** shows images of the signal distribution (1 s after the end of 1 s 60 Hz stimulus) before and after the application of DOI. The quantification of these results shows that no frequency of stimulation altered circuit activity in all three layers of PC (**Figures 4B–D**). To test if prior CRFR_1_ activation could induce a DOI response we first applied CRF and then DOI. Under these conditions DOI produced similar responses to those produced by CRF. In **Figure 5** we show images of responses taken before and after the application of these agonists. In **Figure 5A** the control response is in the left panel while in the middle panel we show the activity of CRF and in the right panel we show the effect of DOI. It is evident that DOI further reduced the effects of the activation of this circuit [*F*(1,9) = 9.82, *p* < 0.01, *n* = 9]. In **Figure 5B** we show the quantitation of the effects of CRF and DOI after CRF in all three layer of PC. These data show that 5-HT_2A/C_R activity is dependent on the prior activation of CRFR_1_. Unlike CRF, the effects of DOI were not reversible within 30 min of “washouts” that were attempted in these experiments.

### The Activity of DOI is Dependent on CRF and 5-HT_2_ Receptor Complex Formation Mediated by a PDZ Domain-Containing Protein

**FIGURE 6:**
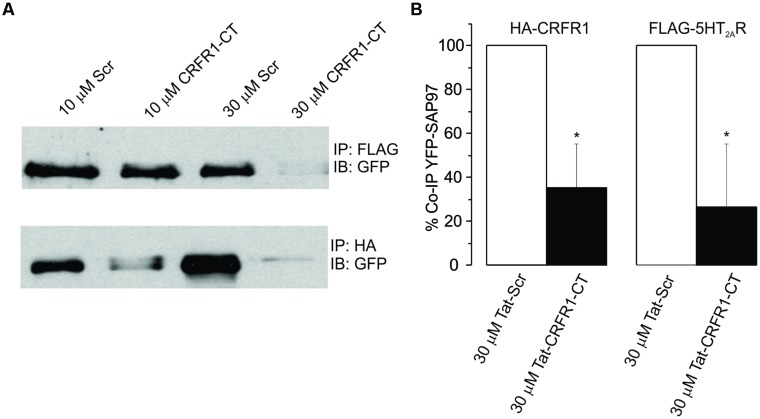
****(A)** HEK293 cells transiently transfected with YFP-SAP97 and either FLAG-5-HT_2A_R or HA-CRFR_1_**. Cells were treated with control or CRFR_1_-CT tat-tagged peptides. YFP-SAP97 co-immunoprecipitation with either FLAG-5-HT_2A_R or HA-CRFR_1_ is significantly inhibited following CRFR_1_-CT treatment. **(B)** Quantitative densitometric analysis of co-immunoprecipitated YFP-SAP97 western blots, ^*^p < 0.05.

**FIGURE 7:**
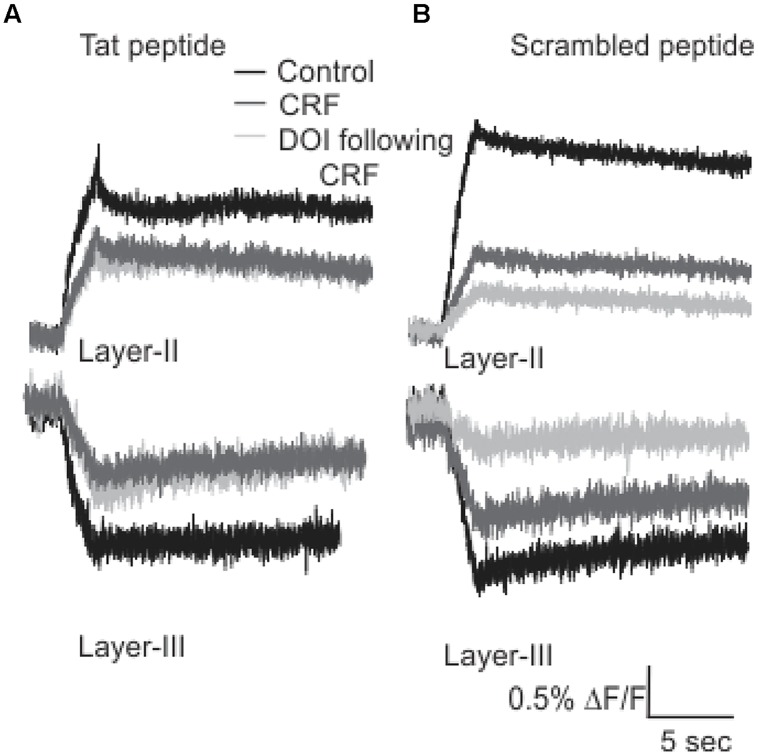
**Tat-CRFR_1_-CT peptide abolished the activity of DOI but not CRF**. Representative traces **(A)** of Layer II (top panel) and Layer III (bottom panel) shows that after the incubation of slices with Tat-CRFR_1_-CT peptide peptide CRF was able to show its usual effects but DOI even applied after CRF failed to show its effect. **(B)** Incubation of slices in scrambled peptide did not prevent the interaction of CRFR_1_ and 5-HT_2A/C_Rs.

It has been previously reported that the activation and subsequent desensitization of CRFR_1_ leads to a complex formation by CRFR_1_ with 5-HT_2_R on endosomes involved in trafficking to the cell membrane (Magalhaes et al., 2010). Therefore we wanted to determine if the DOI effect after CRFR_1_ stimulation may occur through the crosstalk of CRFR_1_ and 5-HT_2A/C_R, which has previously been demonstrated to involve PDZ interactions (Magalhaes et al., 2010). To do this, we utilized a Tat-tagged peptide corresponding to the final 15 amino acids of the CRFR_1_ carboxyl terminus (Tat-CRFR_1_-CT) which includes a class I PDZ-binding motif similar to that found in 5-HT_2A/C_R. This peptide was previously demonstrated to abolish the functional crosstalk between CRFR_1_ and 5-HT_2A/C_R (Magalhaes et al., 2010). Therefore, we investigated whether this peptide could sequester PDZ domain-containing proteins capable of interacting with class I PDZ-binding motifs, thereby preventing their interaction with CRFR_1_ and 5-HT_2A/C_Rs. **Figure 6** shows that 30 μM of Tat-CRFR_1_-CT was sufficient to block the interaction of HA-CRFR_1_ with PDZ domain-containing protein YFP-SAP97 in HEK293 cells. Additionally, the interaction of FLAG-5-HT_2A_R with PDZ domain-containing proteins was inhibited at 30 μM of Tat-CRFR_1_-CT, as evidenced by a lack of interaction between FLAG-5-HT_2A_R and YFP-SAP97 (**Figures 6A,B**). Therefore, Tat-CRFR_1_-CT is capable of blocking the interaction of both CRFR_1_ and 5-HT_2A_Rs with PDZ domain-containing proteins, thereby providing a potential mechanism for the inhibition of receptor crosstalk. Next we determined if the same peptide could prevent the activity of DOI after CRF in brain slices. As a control we also used a scrambled Tat-tagged sequence. The Tat-CRFR_1_-CT peptide was incubated with the slices for 40 min before the application of CRF and DOI following CRF. CRF produced effects comparable to the effects that it produced in the absence of Tat-CRFR_1_-CT while the DOI activity after CRF receptor activation was abolished (**Figure 7**). The scrambled peptide was without effect and DOI was able to further potentiate the activity of CRF. These observations are consistent with interpretation that in order for DOI to have an effect CRFR_1_ must interact with 5-HT_2_Rs through a complex involving PDZ domain-containing proteins.

### The Effects of CRF are Mediated through a Protein Kinase C Dependent Signaling Cascade

**FIGURE 8:**
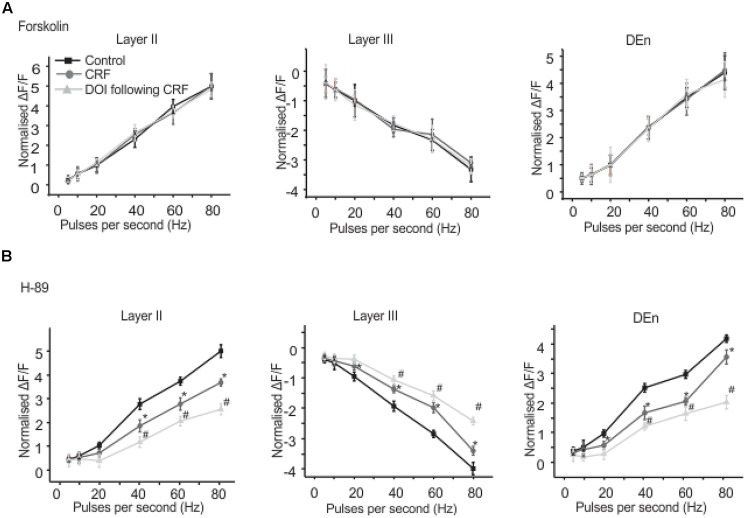
**Activation of adenylate cyclse abolished CRF/DOI activity while antagonism of PKA has no effect**. In **(A)** we show the quantification of the effects of adenylcyclase activation which blocked the effects of CRF/DOI in the Layers II, III, and DEn. **(B)** Shows that the inhibition PKA by H-89 (a PKA antagonist) has no effect on the CRF/DOI responses in all regions. ^*^CRF effect is significantly different compared to control. # DOI following CRF effect is significantly different from CRF ^*^#*p* < 0.05.

**FIGURE 9:**
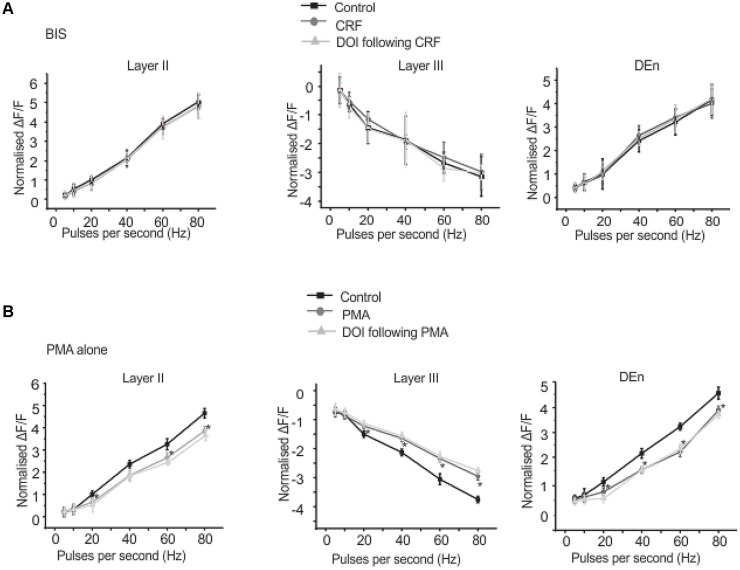
**Corticotrophin-releasing factor receptor 1 activity is mediated through PKC activation**. Application of BIS, a PKC antagonist prevented the effects of CRF and DOI following CRF. In **(A)** we show quantitation of PKC antagonism in the presence of CRF and DOI over the range of stimulation frequencies used to activate the circuit. Application of PMA, a PKC agonist alone mimicked the CRF effects. However, application of DOI following PMA failed to produce further change in the circuit activity. In **(B)** we show quantitation of PKC agonism in the presence of both CRF and DOI over the range of frequencies used to activate the circuit. ^*^p < 0.05. PMA effect is significantly different compared to control.

We found that CRFR_1_ mediates the actions of CRF as antalarmin blocked the actions of CRF. Next we determined which signaling cascade is activated by CRFR_1_ stimulation. Although the CRFR_1_ is usually classified as GPCR that is coupled to Gα_s_ (activation of adenylcyclase), there are several reports that CRFR_1_ signaling can activate Gα_q/11_ cascades as well (Elliott-Hunt et al., 2002; Blank et al., 2003; Wietfeld et al., 2004). To see if a Gα_s_ mediated pathway may be activated by CRFR_1_ simulation we first used the adenylylcyclase activator forskolin (20 μM, for 15–20 min) to see if it would mimic the effects of CRF in all layers of PC. It did not produce effects like CRF (not shown). However, the subsequent application of CRF and/or DOI also had no effect and so activation of adenylcyclase occludes the effects of CRF/DOI (**Figure 8A**). Next we used the PKA antagonist H-89 (10 μM) to see if it could affect the CRF/DOI responses. It had no activity on either the CRF or the DOI response (**Figure 8B**). The responses of CRF and DOI after CRF were still significant in the presence of H-89 [*F*(1,5) = 6.34, *p* < 0.05, *n* = 6]. Thus antagonism of PKA activity does not affect the CRF/DOI responses. Next we applied bisindolylmaleimide-I (BIS; 100 nM), a PKC antagonist to the slices before the application of CRF and DOI following CRF. We found that BIS blocked the effects of CRF and DOI following CRF (**Figure 9A**; *n* = 6). In the next step we applied PMA alone, an activator of PKC to the see if it could mimic the effects of CRF. We observed that PMA (100 nM) produced effects like CRF. However, DOI applied after PMA failed to produce a further change in the PC activity (**Figure 9B**; *n* = 6).

### Single Cell Activity Correlates to VSDI

**FIGURE 10:**
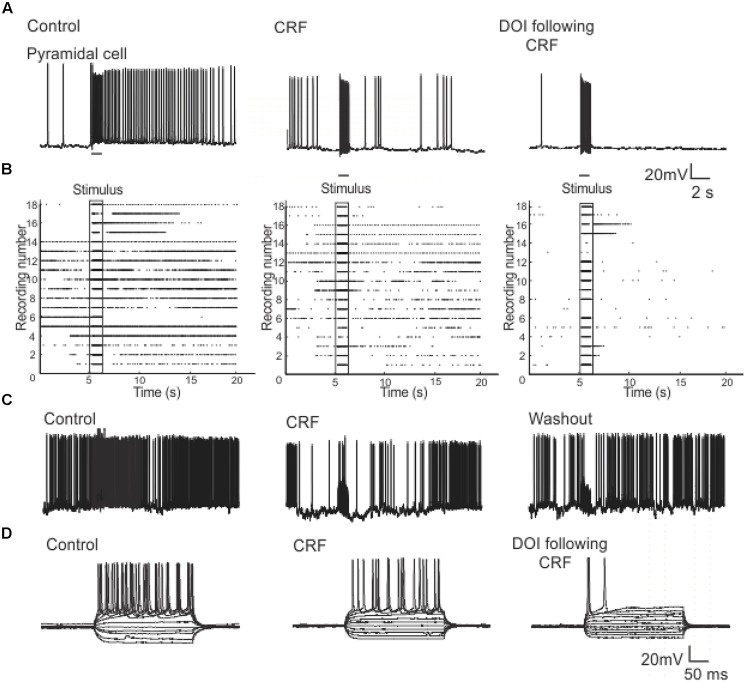
**Patch clamp recordings from Layer II pyramidal cells show that CRF and DOI reduced action potential firing**. In **(A)** a current clamp recording from a pyramidal cell we show that CRF and subsequently DOI attenuated the excitation produced by a 1 s 80 Hz pulse. In **(B)** a raster plot of 18 recordings from Layer II cells shows a summary of these data. In **(C)** like in the VSD recordings the effects of CRF were slowly reversible. In **(D)** a current clamp recording from a typical pyramidal cell shows that the number of action potentials produced by current injection over a range of current steps was reduced by CRF and DOI. This effect was primarily due to increased accommodation with no prominent change in firing frequency (see text for a more complete description).

In order to understand how CRF/DOI may affect the activity of individual cells we conducted whole-cell patch clamp recordings in the Layers II and III of PC. To examine the responses in the Layer II we stimulated LOT using 80 Hz trains which were identical to stimuli used for VSD imaging. The response to this train was reduced by CRF (**Figure 10A**) even though each stimulus generated an action potential during the train (not shown). Thus CRF does not seem to have any effect on the efficacy of the stimulus only the response to them. The addition of DOI after CRF further reduced the efficacy of these stimuli. In **Figure 10B** we show a raster plot of 18 recordings done from pyramidal cells showing that both CRF and DOI reduced the activity induced by LOT stimulation (*n* = 10). Like that observed in the VSDI recordings the effects of CRF were reversible after about 15–20 min of washing (**Figure 10C**), but we saw no reversibility within the same time frame for DOI after CRF applications. Finally, we also found that CRF and DOI reduced the excitability of pyramidal cells as equivalent current pulses were unable to generate same activity during a 500 ms pulse of current (**Figure 10D**). In control recordings at two times the threshold current required to induce an action potential (I_2t_) the average interspike frequency of the train was 44.8 ± 1.1 Hz while in CRF and DOI this was 39.0 ± 2.0 Hz (*p* < 0.01) and 42.9 ± 1.3 Hz respectively (*p* > 0.05 compared to control). Thus the average frequency of trains generated was not largely changed. However, the number of action potentials generated by this current injection was greatly reduced (Control 11 ± 1, CRF 6 ± 1, and DOI 2 ± 1 *p*’s < 0.001 for all comparisons). Thus the pyramidal cells generated action potentials at similar or slightly reduced frequencies but the cells accommodated to the stimulus generating fewer action potentials.

**FIGURE 11:**
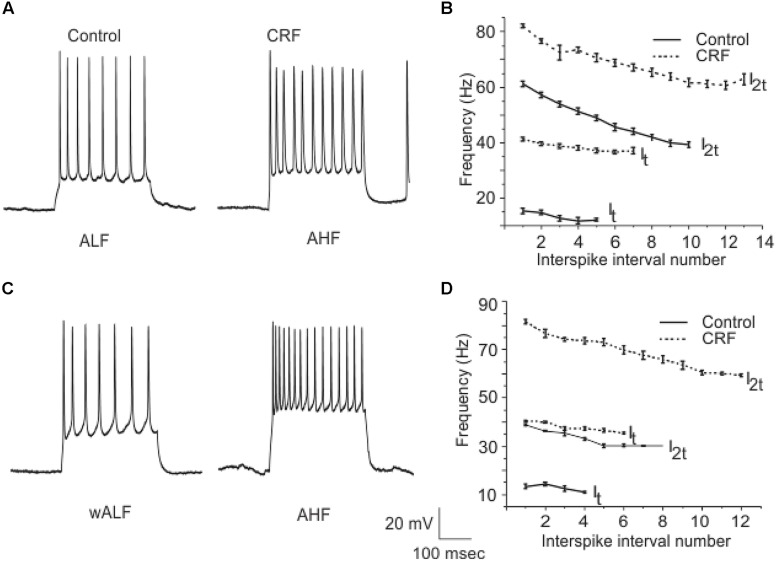
**A subpopulation of interneurons from Layer III increased their firing frequency after the application of CRF**. In **(A)** we show an example of ALF interneuron that converted to AHF firing pattern after the application of CRF. The graphs in **(B)** show the average of firing frequency against interspike interval number from 14 ALF at one times threshold and two times threshold (I_t_ and I_2t_ respectively). **(C)** Shows an example of a wALF (weakly adapting low frequency) interneuron that converted to AHF type after the application of CRF. A plot of the average of firing frequency against interspike interval number from the five ALF interneurons that converted to AHF after the application of CRF is shown in **(D)**.

**FIGURE 12:**
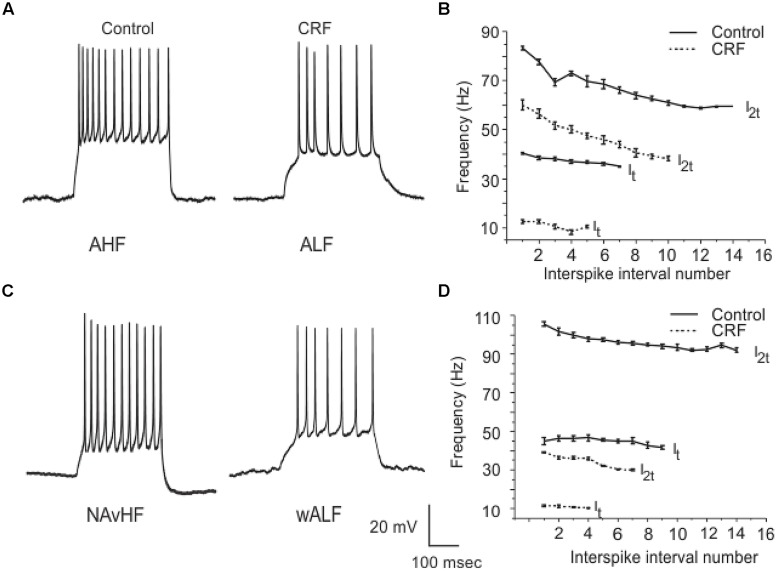
**Another subpopulation of interneurons from Layer III reduced their firing frequency after the application of CRF**. In **(A)** an example of AHF interneuron that converted to ALF phenotype after the application of CRF is shown. The plots in **(B)** show how the average firing frequency versus interspike interval number was changed in the six interneurons where this occurred at one times threshold and two times threshold (I_t_ and I_2t_ respectively). Similarly in **(C)** we show an example of a NAvHF interneuron that converted to the wALF phentotype after CRF application. **(D)** Shows the quantification of the firing frequency against interspike interval number for five NAvHF interneurons.

Corticotropin-releasing factor had variable and highly complex effects on the excitability of cells located in Layer III which are almost exclusively GABAergic interneurons. In contrast to pyramidal cells DOI had no effect on any recordings done in this cell layer. We have previously shown that interneurons in Layer III fire with five well-defined and differing spike patterns (Gavrilovici et al., 2012). The naming of these firing patterns were adopted from the nomenclature defined in Ascoli et al. (2008). Here we found that CRF altered the spiking patterns of some, but not all these subtypes. There were two types of outcomes on these interneurons. One response converted low frequency firing patterns to high frequency ones. While the second type of response showed a conversion from a high frequency spiking patterns to low. In some recordings there was no apparent effect of CRF. In **Figure 11A** we show an example of the conversion of an ALF interneuron (<50 Hz average firing frequency at two times threshold (I_2t_); also see methods for description of classifications) to an AHF phenotype (>50 Hz at I_2t_). All interneurons having this initial phenotype responded in this manner (Control: 44.0 ± 0.1; CRF: 62.3 ± 0.6 Hz, *p* < 0.001, *n* = 14). The graph in **Figure 11B** shows the averaged interspike frequency versus spike interval relationship for all 14 recordings. One line shows the relationship at the threshold (I_t_) where a train of action potentials is elicited, while the other shows the relation at I_2t_. It is evident that CRF produced a dramatic change in the input/output responses of these cells. Similarly those cells that had the weakly ALF phenotype (wALF) all converted to an AHF pattern (**Figure 11C**; Control: 29.1 0 ± 0.6; CRF: 69.1 ± 0.8 Hz, *p* < 0.001, *n* = 6). The average change in the input/output for this population is shown in **Figure 11D**. The second type of responses were more variable. Interneurons that initially fired with an AHF phenotype sometimes converted to ALF pattern (**Figures 12A,B**; Control: 62.9 ± 0.9; CRF: 43.9 ± 1.0 Hz, *p* < 0.001, *n* = 6) but another cohort did not change (*n* = 7; not shown). NAvHF interneurons (>100 Hz) similarly had variable responses; some did not change (*n* = 4) while another group converted to the weakly adapting low frequency interneuron phenotype (wALF; **Figures 12C,D**, Control: 101.3 ± 1.0; CRF: 28.1 ± 0.9 Hz, *p* < 0.001, *n* = 5). The fifth pattern that we identified (Gavrilovici et al., 2012), strongly ALF, which occurred in less than 5% of the 205 recordings, was not seen in 38 recordings we did here and so we are unsure as to the effects of CRF on these cells.

## Discussion

Disturbances in homeostasis due to stressors are thought to mitigate the onset of depression or anxiety (de Kloet et al., 2005). The physiological mechanisms following stressful stimuli involve various brain regions, such as amygdala, hippocampus and nucleus accumbens. These areas interact and integrate their responses by releasing neuromodulators that regulate stressor reactions (Mora et al., 2012). The importance of these brain regions during stress and anxiety has been extensively studied (McEwen, 2007). The PC, a part of the limbic region that is interconnected with amygdale, hippocampus, and prefrontal cortex (Loscher and Ebert, 1996), has not been extensively studied to determine how it may respond to stressors. Odor perception can induce stressor responses which are highly context dependent (Kadohisa, 2013; Krusemark et al., 2013) and therefore it is reasonable to hypothesize the PC could be modulated by neurochemical processes depending on the nature of the stressor. In this context, we have shown here that CRF and 5-HT receptors, which are implicated in stress, anxiety and depression in many regions (Aghajanian and Davis, 1975; Kahn et al., 1988; Eison, 1990; Davis, 1992; Arborelius et al., 1999; Naughton et al., 2000; Muller et al., 2003; Merali et al., 2004; Risbrough and Stein, 2006) have profound effects in the PC. What is surprising is that although CRF and 5-HT_2_R activation is known to be anxiogenic, they dampened the overall activity of the PC. Thus deactivation of PC would seem to be the usual response to these ligands in this particular brain region. As the primary function of the PC is olfaction this may in fact be a reflexive response to dampen output to the amygdala from the PC which is presumably already being, through prefrontal or other pathways, activated by the perceived threat. Our data also indicate that serotonergic neurotransmission on 5-HT_2_R receptors, in the stimulation paradigm used here, when CRF is released (presumably from the central amygdala or local CRF containing neurons). Thus, it would seem that amygdala activation is required for this CRF/DOI interaction to occur.

The effects of DOI were dependent on prior CRFR_1_ activation requiring the formation – CRFR_1_ – 5-HT_2_R complex. However, the interaction of CRFR_1_ and 5-HT_2A/C_Rs appears to only occur on pyramidal cells as the interneurons of Layer III were unaffected by DOI application. As pyramidal cells express both 5-HT_2A/C_Rs we are unsure what subtype(s) are being trafficked. Although the application of PMA mimicked CRF, activation of PKC did not induce DOI responses (like CRF). This outcome fits with the interpretation that the DOI activity is dependent on CRFR_1_ activation and presumably CRFR_1_ endocytosis. So although PKC activation dampened excitability, DOI could not add to this effect as CRFR_1_ activation had been bypassed.

Corticotropin-releasing factor activity seems to vary with brain region and cell type. Another study using VSDI demonstrated that CRF enhanced hippocampal excitation (von et al., 2011) and several patch clamp studies reported that CRF was able to facilitate action potential firing in hippocampus (Aldenhoff et al., 1983; Hollrigel et al., 1998; Blank et al., 2003). CRF has also been shown to depress excitatory neurotransmission in the hippocampal formation (Sheng et al., 2008), but we found no evidence of this here as LOT stimulation was unaffected by CRF. It has also been shown that in the prefrontal cortex CRF decreased the excitability of pyramidal cells by enhancing GABA release (Tan et al., 2004). Although we did not measure GABA release here, this outcome seems unlikely. The NAvHF interneurons whose excitability was reduced densely innervates Layer II pyramidal cells and therefore a reduction GABA release would be predicted. The reason for these highly variable outcomes throughout the brain is unclear. However, these outcomes cannot be explained by CRF interacting with differing CRFRs. It is more likely that divergent actions of CRF are, at least, in part due to divergent signaling pathways as other studies have shown that CRFR_1_ can activate either Gα_s_ or Gα_q/11_ signaling. Elliot-Hunt and co-workers reported that CRF activated PKA and MAPK signaling pathways in hippocampus (Elliott-Hunt et al., 2002). In another study, Sheng et al. (2008) observed, in cultured hippocampal neurons, inhibition of PKC abolished CRF regulation of NMDA currents. (Tan et al., 2004) showed that forskolin did not mimic the actions of CRF but PKC activation did; observations that are identical to ours. Thus it seems that CRF signally is promiscuous varying from cell to cell and region to region. Interestingly we found that activation of adenylcyclase by forskolin- occluded the effects of CRF. The mechanism by which this occurs is unclear but may involve heterologous desensitization of the CRFR_1_ itself as a consequence of forskolin mediated activation of PKA. As we have found no evidence CRF signals through differing cascades here (although it cannot be ruled out), it seems more likely that the CRFR_1_ activation is linked to divergent effector cascades that alter neuron function.

The most profound effect of the CRFR_1_/5-HT_2A/C_R interaction is the dampening of the Layer II excitability. These likely accounts for the reduced activation the circuit with the highly variable effects of CRF on interneurons in Layer III modulating the overall response. Although, it would also seem reasonable to conclude that the reduced excitability of the majority of interneurons would also enhance the dampening of circuit activation. Nevertheless, the fact that the activity of CRF in Layer III varied changing from one functionally distinct interneuron subtype to another argues for more subtle modulatory roles for CRF within this circuit. Given that an important function of interneurons is to control network synchrony this suggests that CRF in Layer III modulates the firing patterns of the PC and may also be important for governing the impact of circuit activation. This variability in CRF effects has been observed before; when administered intracerebroventricularly it produced both inhibitory and excitatory effects on dorsal raphae nucleus neuron firing rate. Both effects were mediated through CRFR_1_ as antalarmin attenuated CRF’s activity (Kirby et al., 2000). So in sum our data suggest that the most salient outcome of CRF/DOI activity is to reduce the Layer II activity which then attenuates the disinhibition.

5-HTR activation also has been shown elsewhere to produce heterogeneous effects in many brain regions. Here, we show DOI reduced PC pyramidal cell activity but had no effect on the interneurons in Layer III. By contrast activation of 5-HT_1C_R (subsequently renamed 5-HT_2C_R) receptors in PC produced increased pyramidal cell activity (Sheldon and Aghajanian, 1990). In same study activation of 5-HT_2_Rs in PC increased the occurrence of spontaneous inhibitory post-synaptic potentials (IPSP’s) on pyramidal cells. This was shown to be due to increased firing of interneurons located in the border between Layers II and III. Initially which subtype of 5-HT_2_R was activated was not known as the 5-HT was used as the agonist and a non-selective 5-HT_2_R antagonist was used to block this effect. Later it was shown that this effect was mediated by the 5-HT_2A_R and not 5-HT_2C_R (Marek and Aghajanian, 1994). We found that activation of the 5-HT_2A/C_Rs on pyramidal cells decreased excitability although this required previous CRFR_1_ activation. This argues that the effects are mediated by 5-HT_2A/C_Rs, consistent with the findings of Magalhaes et al. (2010). We also found that DOI had no effect on interneuron spiking patterns. These results are surprising as 5-HT_2A/C_Rs are expressed in this layer and one might expect activation to alter the activity of cells in this layer. Therefore it would seem that the increased interneuron activity observed by Aghajanian and coworkers is not due to changes in their intrinsic excitability and likely represents increased excitatory drive onto these interneurons or alteration in the neurotransmitter release mechanisms.

In summary, this study shows how neuronal activity through PC layers is altered by the activation of CRFR_1_ and 5-HT_2A/C_Rs. To emphasize it is surprising that two neurotransmitters that heighten anxiety would reduce excitability in a circuit which has strong connections to hippocampus, amygdala and prefrontal cortex. This may represent a behavioral feedback mechanism to blunt odor induced stressor responses. What is also striking is the highly complex and varied nature of the CRFR_1_ effects on the differing cell populations. The use of the selective Tat-CRFR_1_-CT peptide indicates these effects of DOI are mediated through an interaction between CRFR_1_/5-HT_2A/C_Rs with one or multiple PDZ domain-containing proteins (like SAP97), but it doesnot rule out other similar interactions may occur with other receptor subtypes. Finally, it seems CRFR_1_ may signal through differing G protein signaling cascades and so it may be possible under differing conditions, epilepsy, chronic stress or perhaps head injury, that these pathways may be plastic and modulation of this network and others may be dynamic.

## Conflict of Interest Statement

The authors declare that the research was conducted in the absence of any commercial or financial relationships that could be construed as a potential conflict of interest.

## Abbreviations

AHF: adapting high frequency
ALF: adapting low frequency
CRF: corticotrophin-releasing factor
CRFR_1_: corticotrophin-releasing factor receptor 1
DOI: dimethoxy-4-iodoamphetamine
5-HT_2A/C_R: 5-hydroxytrytamine _2A/C_ receptor
NavHF: non-adapting very high frequency
PC: piriform cortex.
